# Effect of leptin infusion on insulin sensitivity and lipid metabolism in diet-induced lipodystrophy model mice

**DOI:** 10.1186/1476-511X-7-8

**Published:** 2008-03-18

**Authors:** Koji Nagao, Nao Inoue, Yoko Ujino, Kouki Higa, Bungo Shirouchi, Yu-Ming Wang, Teruyoshi Yanagita

**Affiliations:** 1 Department of Applied Biochemistry and Food Science, Saga University, Saga 840-8502, Japan; 2 College of Food Science and Engineering, Ocean University of China, Qingdao 266003, China

## Abstract

**Background:**

Lipodystrophies are rare acquired and genetic disorders characterized by the complete or partial absence of body fat with a line of metabolic disorders. Previous studies demonstrated that dietary conjugated linoleic acid (CLA) induces hepatic steatosis and hyperinsulinemia through the drastic reduction of adipocytokine levels due to a paucity of adipose tissue in mice and the pathogenesis of these metabolic abnormalities in CLA-fed mice is similar to that in human lipodystrophy. The present study explores the effect of leptin infusion on the pathogenesis of diet-induced lipodystrophy in mice. C57BL/6N mice were assigned to three groups: (1) mice were fed a semisynthetic diet supplemented with 6% corn oil and infused PBS intraperitoneally (normal group), (2) mice were fed a semisynthetic diet supplemented with 4% corn oil plus 2% CLA and infused PBS intraperitoneally (lipodystrophy-control group), and (3) mice were fed a semisynthetic diet supplemented with 4% corn oil plus 2% CLA and infused recombinant murine leptin intraperitoneally (lipodystrophy-leptin group). All mice were fed normal or lipodystrophy model diets for 4 weeks and were infused intrapeneally 0 or 5 *μ*g of leptin per day from third week of the feeding period for 1 week.

**Results:**

The results indicate that leptin infusion can attenuate hepatic steatosis and hyperinsulinemia through the reduction of hepatic triglyceride synthesis and the improvement of insulin sensitivity in diet-induced lipodystrophy model mice.

**Conclusion:**

We expect the use of this model for clarifying the pathophysiology of lipodystrophy-induced metabolic abnormalities and evaluating the efficacy and safety of drug and dietary treatment.

## Background

Recent advances in molecular and cell biology have shown that adipose tissue not only stores excess energy in the form of fat, but also secretes physiologically active substances called adipocytokines [1]. In obesity, it is well known that adipocytes, cells of adipose tissues, are increased and enlarged, and they secrete excess amounts of inflammatory adipocytokines, such as tumor necrosis factor-alpha [2] and monocyte chemoattractant protein-1 [3]. This induces insulin resistance, hyperinsulinemia, and fatty liver [4,5]. On the other hand, it is reported that the deficiency of adipocytes also induces type-2 diabetes due to a paucity of normally functioning adipocytokines such as leptin [6] and adiponectin [7,8]. This symptom is known as lipodystrophy in humans. Lipodystrophies are rare acquired and genetic disorders characterized by the complete or partial absence of body fat with a line of metabolic disorders [9,10]. Recent reports indicated that the clinical treatment of HIV-infected patients by using HIV-1 protease inhibitors also induces acquired lipodystrophy [10].

**Figure 1:**
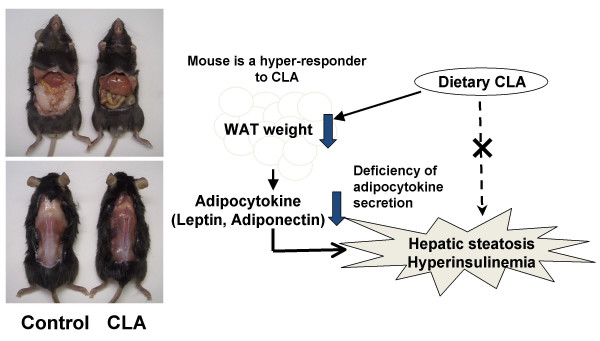
Scheme showing possible mechanisms of CLA-induced lipodystrophy.

To understand the pathophysiology of lipodystrophy and evaluate the efficacy and safety of clinical treatments, several transgenic mouse models that mimic the features of lipodystrophy, such as aP2-SREBP-1c mouse [11-13] and A-ZIP/F1 mouse [14-16], have been established. Additionally, it has been reported that feeding of conjugated linoleic acid (CLA), a group of positional and geometric isomers of linoleic acid, with a low-fat diet also induces lipodystrophy, characterized by an increase in hepatic lipid content concomitant with a decrease in body fat mass in mice [17,18]. It has been suggested that lipodystrophy may occur in mice because they are too sensitive to the CLA-induced reduction in body fat [19,20]. We previously reported that short-term feeding of CLA decreased weights of adipose tissues and hepatic lipid levels without inducing adverse effects in mice [21]. Tsuboyama-Kasaoka, Miyazaki, Kasaoka, and Ezaki [22] also reported that increasing the amount of fat in a CLA-supplemented diet substantially reduces the lipodystrophy effect. These results indicate that dietary CLA induces fatty liver and hyperinsulinemia through the drastic reduction of adipocytokine levels due to a paucity of adipose tissue, but not through the direct induction of hepatic lipid synthesis and insulin resistance (Figure 1). Because the pathogenesis of these metabolic abnormalities in CLA-fed mice is similar to that in human lipodystrophy, we expect the use of CLA-fed mice as a diet-induced lipodystrophy model.

In the present study, we investigated the effects of leptin infusion on insulin sensitivity and lipid metabolism in diet-induced lipodystrophy model mice. Previous studies demonstrated that leptin treatment attenuated insulin resistance in genetically diabetic mice (such as *ob/ob *mice and MKR mice) [23,24] and in lipodystrophy model transgenic mice (such as aP2-SREBP-1c mice) [25]. In addition, Tsuboyama-Kasaoka, Takahashi, Tnemura, Kim, Tnage, Okuyama, Kasai, Ikemoto, and Ezaki [26] showed preliminary data indicating that leptin infusion lowers the levels of serum insulin and attenuates hepatocyte fat deposition in CLA-fed lipodystrophy model mice. To clarify the precise effect of leptin infusion, we measured hepatic enzyme activities in relation to lipid metabolism and tested insulin sensitivities in these model mice.

## Results and Discussion

### Effect of leptin infusion on growth parameters in diet-induced lipodystrophy model mice

**Figure 2:**
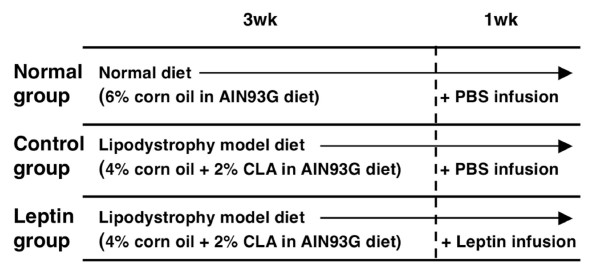
**Experimental design.** Mice were fed normal diet (normal group) or lipodystrophy model diets (control group and leptin group) for 4 weeks and were infused intrapeneally 0 or 5 *μ*g of leptin per day for the final week of the 4-week feeding period.

The experimental design is indicated in Figure 2. Table 1 shows the effect of leptin infusion on growth parameters in diet-induced lipodystrophy model mice. Although there was no significant difference in final body weight or food intake among groups, CLA-containing lipodystrophy model diets significantly increased the liver weight of mice, as has been reported elsewhere [17-20]. Leptin infusion alleviated, but not significantly, hepatomegaly in diet-induced lipodystrophy model mice. Weights of waist subcutaneous and abdominal (perirenal, epididymal, and omental) white adipose tissue (WAT) were significantly decreased in lipodystrophy model mice, and there was no significant effect of leptin infusion on WAT weights in this model.

**Table 1:** Effect of leptin infusion on growth parameters in C57BL/6N mice

		Lipodystrophy model	
		
	Normal	Control	Leptin
Final body weight (g)	21.7 ± 0.5	22.1 ± 0.3	21.3 ± 0.2
Food intake (g)	61.2 ± 1.3	58.4 ± 2.1	59.6 ± 1.2
Liver (g/100 g body weight)	4.11 ± 0.05 ^a^	6.47 ± 0.77 ^b^	5.65 ± 0.29 ^b^
Whit adipose tissue (g/100 g body weight)			
Epididymal	1.87 ± 0.22 ^a^	0.290 ± 0.029 ^b^	0.228 ± 0.030 ^b^
Perirenal	0.836 ± 0.104 ^a^	0.199 ± 0.018 ^b^	0.178 ± 0.018 ^b^
Omental	1.13 ± 0.09 ^a^	0.652 ± 0.022 ^b^	0.740 ± 0.029 ^b^
Subcutaneous	1.95 ± 0.16 ^a^	0.329 ± 0.014 ^b^	0.344 ± 0.017 ^b^

^a, b ^Different superscript letters show significant difference at *P *< 0.05.

### Effect of leptin infusion on hepatic triglyceride metabolism in diet-induced lipodystrophy model mice

**Figure 3:**
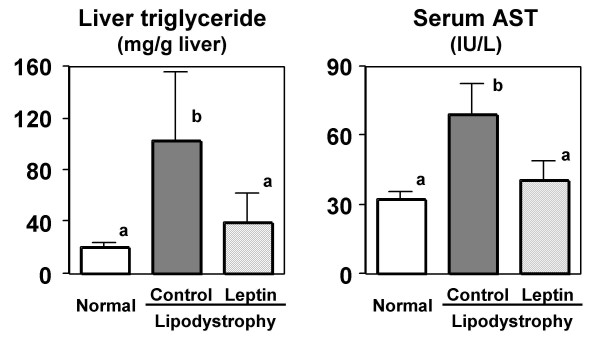
**Effect of leptin infusion on hepatic triglyceride levels and serum alanine aminotransferase activities in diet-induced lipodystrophy model mice.** Mice were fed normal or lipodystrophy model diets for 4 weeks and were infused intrapeneally 0 or 5 *μ*g of leptin per day f for the final week of the 4-week feeding period. Values are expressed as mean ± SE. ^a, b^Different letters show significant differences at *P *< 0.05. AST, alanine aminotransferase.

Figure 3 shows hepatic triglyceride levels and serum aspartate aminotransferase (AST) levels of C57BL/6N mice at the end of the experiment. The hepatic triglyceride level in mice fed the lipodystrophy model diet was 5-fold that in the mice fed a normal diet, and 1-week infusion of leptin resulted in a 62% attenuation of triglyceride accumulation in the liver. The activities of AST, one of the hepatic injury markers, in the serum of lipodystrophy model mice were markedly increased because of the development of hepatic steatosis. Leptin infusion to the rats fed lipodystrophy model diets, however, resulted in a 41% decrease of AST levels consistent with the attenuation of hepatic triglyceride accumulation.

**Figure 4:**
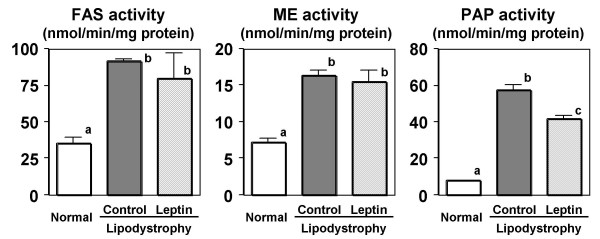
**Effect of leptin infusion on activities of enzymes related to lipid metabolism in the liver of diet-induced lipodystrophy model mice.** Mice were fed normal or lipodystrophy model diets for 4 weeks and were infused intrapeneally 0 or 5 *μ*g of leptin per day for the final week of the 4-week feeding period. Values are expressed as mean ± SE. ^a, b^Different letters show significant differences at *P *< 0.05. FAS, fatty acid synthase; ME, malic enzyme; PAP, phosphatidate phosphohydrolase.

Takahashi, Kushiro, Shinohara, and Ide [27] demonstrated that CLA increases the activity and mRNA levels of hepatic lipogenic enzymes; they suggested that enhanced lipogenesis is a principal mechanism of CLA-induced hepatic steatosis in mice. In this study, activities of fatty acid synthase (FAS) and malic enzyme (ME) were increased in the liver of mice fed the lipodystrophy model diet, as has been reported previously (Figure 4). Leptin infusion, however, did not change those lipogenic enzyme activities in diet-induced lipodystrophy model mice. We also measured the activities of phosphatidate phosphohydrolase (PAP), the key enzyme in the regulation of TG *de novo *synthesis [28] (Figure 4). The lipodystrophy model diet increased the activities of the membrane-bound forms of Mg^2+^-dependent PAP, but these activities were significantly suppressed by the leptin treatment in C57BL/6N mice. Therefore we supposed that hepatic steatosis induced by the lipodystrophy model diet was attenuated by leptin infusion partly through the suppression of triglyceride synthesis.

### Effect of leptin infusion on adipocytokine levels and insulin sensitivities in diet-induced lipodystrophy model mice

**Figure 5:**
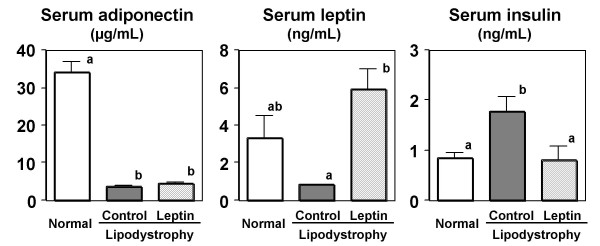
**Effect of leptin infusion on serum adipocytokines and insulin levels in diet-induced lipodystrophy model mice.** Mice were fed normal or lipodystrophy model diets for 4 weeks and were infused intrapeneally 0 or 5 *μ*g of leptin per day for the final week of the 4-week feeding period. Values are expressed as mean ± SE. ^a, b^Different letters show significant differences at *P *< 0.05.

**Figure 6:**
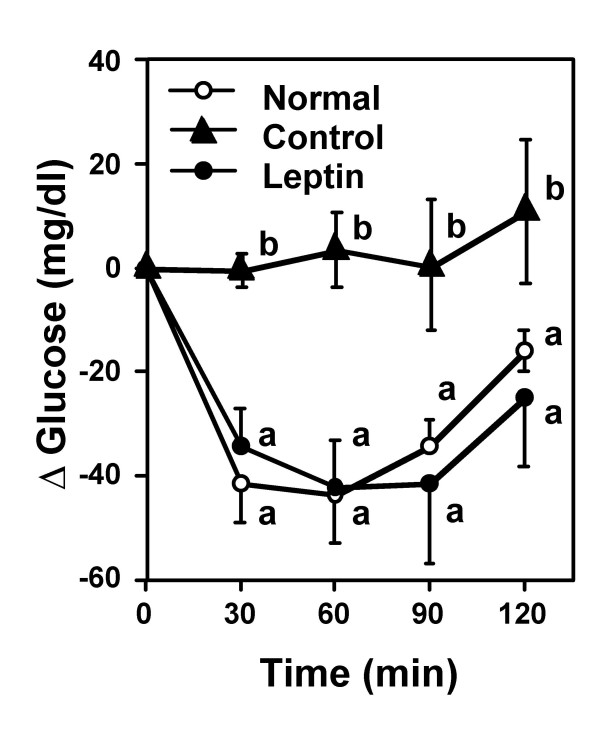
**Effect of leptin infusion on insulin sensitivities in diet-induced lipodystrophy model mice.** Mice were fed normal or lipodystrophy model diets for 4 weeks and were infused intrapeneally 0 or 5 *μ*g of leptin per day for the final week of the 4-week feeding period. Blood glucose was measured at the indicated time points. Values are expressed as mean ± SE. Means at a time without a common letter differ at *P *< 0.05.

As shown in Figure 5, serum levels of adiponectin and leptin were drastically decreased in mice fed the CLA-containing lipodystrophy model diet, as previously reported [17-20]. Adiponectin and leptin are both abundantly secreted from adipose tissue and have several physiological functions, including the regulation of insulin sensitivity in humans and animals. Therefore it has been reported that the deficiency of adipocytokine secretion induced by a paucity of adipose tissue would be a cause of lipodystrophy, which is characterized by a severe insulin resistance and leads to hyperinsulinemia and hepatic steatosis [19,20]. In the present study, hepatic steatosis and hyperinsulinemia were alleviated by leptin infusion in mice fed the lipodystrophy model diet, concomitant with the alleviation of leptin deficiency (Figure 5). However, leptin infusion did not affect adiopnectin levels, compared with those seen with PBS injection in mice fed the lipodystrophy model diet. As shown in Figure 6, the insulin-mediated glucose lowering effect was impaired in mice fed the lipodystrophy model diet, consistent with serum insulin levels (Figure 5). However, the insulin resistance induced by the lipodystrophy model diet was markedly alleviated by leptin infusion in diet-induced lipodystrophy model mice (Figure 6). These results suggest that leptin treatment attenuates hepatic steatosis and hyperinsulinemia through the alleviation of insulin resistance in the diet-induced lipodystrophy model, as has been shown in various lipodystrophy models [23-25]. Although previous reports demonstrated that treatment with rosiglitazone (an insulin sensitizer) can improves insulin resistance concomitant with an increase of adiponectin level in CLA-fed mice [29,30], our results suggest that leptin replacement is sufficient to alleviate hepatic steatosis and hyperinsulinemia in this diet-induced lipodystrophy model.

## Conclusion

The present study explored the effect of leptin infusion on the pathogenesis of diet-induced lipodystrophy in mice. The results indicate that leptin infusion can attenuate hepatic steatosis and hyperinsulinemia through the reduction of hepatic triglyceride synthesis and the improvement of insulin sensitivity in diet-induced lipodystrophy model mice. We expect the use of this model for clarifying the pathophysiology of lipodystrophy-induced metabolic abnormalities and evaluating the efficacy and safety of drug and dietary treatment.

## Methods

### Animals and diets

All aspects of the experiment were conducted according to the guidelines provided by the Ethical Committee of Experimental Animal Care at Saga University. C57BL/6N mice (Kyudo Co., Ltd., Saga, Japan) were housed individually in metal cages in a temperature-controlled room (24°C) under a 12-hour light/dark cycle. Mice were assigned to three groups (3–6 mice each): (1) mice were fed a semisynthetic diet supplemented with 6% corn oil and infused PBS (Gibco, Tokyo, Japan) intraperitoneally (normal group), (2) mice were fed a semisynthetic diet supplemented with 4% corn oil plus 2% CLA and infused PBS intraperitoneally (lipodystrophy-control group), and (3) mice were fed a semisynthetic diet supplemented with 4% corn oil plus 2% CLA and infused recombinant murine leptin (5 *μ*g/day, PeproTech EC, London, United Kingdom) intraperitoneally (lipodystrophy-leptin group). The semisynthetic diets were prepared according to recommendations of the AIN-93G [31] and contained (in weight %) casein, 20; fat, 6; alpha-cornstarch, 13.2; vitamin mixture (AIN-93™), 1; mineral mixture (AIN-93G™), 3.5; L-cystein, 0.3; choline bitartrate, 0.25; cellulose, 5; sucrose, 10; tert-buthyhydroquinone, 0.0014; and beta-cornstarch, 40.7486. The mice received the diets ad libitum using Rodent CAFE (KBT Oriental Co. Ltd., Saga, Japan) for 4 weeks. The mice were killed by exsanguination of the heart, and serum was separated from the blood. Liver and WATs (perirenal, epididymal, omental, and waist subcutaneous) were also excised for analysis.

### Analysis of hepatic triglyceride and serum parameters

Liver lipids were extracted according to the method of Folch, Lee, and Sloane-Stanley [32], and the concentrations of triglyceride were measured using the methods of Fletcher [33]. Serum insulin, adiponectin, and leptin levels were measured using commercial mouse ELISA kits (Shibayagi Co. Ltd., Gunma, Japan; Otsuka Pharmaceutical Co. Ltd., Tokyo, Japan; and Morinaga Co. Ltd., Yokohama, Japan, respectively). Activities of AST in serum were measured using commercial enzyme assay kits (Wako Pure Chemicals, Tokyo, Japan).

### Measurement of hepatic enzyme activities

A piece of liver was homogenized insix volumes of a 0.25-M sucrose solution that contained 1 mM EDTA in a 10-mM Tris-HCL buffer (pH 7.4). Fractions of cytosol and microsomes were obtained as previously described [34]. The protein concentration was determined according to the method of Lowry, rosebrough, Farr, and Randall [35], with bovine serum albumin used as the standard. The enzyme activities of ME (EC 1.1.1.40) [36] and FAS (EC 2.3.1.85) [37] in the liver cytosol fraction and phosphatidate phosphohydrolase (EC 3.1.3.4) [38] in the liver microsomal fraction were determined as described.

### Insulin tolerance test

At the end of the feeding period, human insulin (Humulin R; Eli Lilly Japan K.K., Kobe, Japan) was injected intraperitoneally (0.75 mU/g body weight) to all mice. Blood glucose was measured on samples obtained from tail tip before and 30, 60, 90, and 120 min after insulin injection. Blood glucose concentrations were measured using the GLUCOCARD™ G meter (Arkray, Kyoto, Japan).

### Statistical analysis

All values are expressed as means ± SE. Data were analyzed by one-way ANOVA, and all differences were inspected by Duncan's new multiple-range test [39]. Differences were considered to be significant at *P *< 0.05.

## List of abbreviations

AST, aspartate aminotransferase; CLA, conjugated linoleic acid; FAS, fatty acid synthase; ME, malic enzyme; PAP, phosphatidate phosphohydrolase; WAT, white adipose tissue.

## Competing interests

The author(s) declare that they have no competing interests.

## Authors' contributions

KN contributed in planning, experimental work, analysis and publication of results. NI contributed in planning, experimental work, analysis, and discussion. YU contributed in experimental work and analysis. KH contributed in experimental work and analysis. BS contributed in planning of the experiment and in discussion of results. YMW contributed in planning of the experiment and in discussion of results. TY contributed in planning of the experiment, discussion of results, and providing funding for the study. All authors read and approved the final manuscript.
